# Appendiceal goblet cell carcinoma has marginal advantages from perioperative chemotherapy: a population-based study with an entropy balancing analysis

**DOI:** 10.1007/s00423-023-02791-x

**Published:** 2023-01-25

**Authors:** Claudio Ricci, Davide Campana, Carlo Ingaldi, Giuseppe Lamberti, Laura Alberici, Valentina Tateo, Giovanni Castagna, Gianluca Ricco, Fulvio Calderaro, Deborah Malvi, Francesca Rosini, Riccardo Casadei

**Affiliations:** 1 Division of Pancreatic Surgery, IRCCS Azienda Ospedaliero-Universitaria Di Bologna, Via Albertoni 15, Bologna, Italia; 2 Department of Internal Medicine and Surgery (DIMEC), Alma Mater Studiorum, University of Bologna, Bologna, Italy; 3 Division of Oncology, IRCCS Azienda Ospedaliero-Universitaria Di Bologna, Via Albertoni 15, Bologna, Italia; 4 Department of Specialized,Diagnostic and Experimental Medicine (DIMES), Alma Mater Studiorum, University of Bologna, Bologna, Italy; 5 Division of Pathology, IRCCS Azienda Ospedaliero-Universitaria Di Bologna, Via Albertoni 15, Bologna, Italia

**Keywords:** Goblet cell carcinoma, Appendiceal neoplasm, Chemotherapy, Surgical resection

## Abstract

**Purpose:**

The aim is to clarify the use of perioperative chemotherapy in resectable goblet cell carcinoma (GCC).

**Methods:**

A retrospective study was carried out based on the Surveillance, Epidemiology, and End Results study. The population was divided: into patients who received only radical surgery (group A) and those who received radical surgery plus chemotherapy (group B). An entropy balancing was carried out to correct the imbalance between the two groups. Two models were generated. Model 1 contained only high-risk patients: group B and a “virtual” group A with similar characteristics. Model 2 included only low-risk patients: group A and “virtual” group B with identical attributes. The efficacy of entropy balancing was evaluated with the *d* value. The overall survival was compared and reported with Hazard Ratio (HR) within a confidence interval of 95% (95 CI).

**Results:**

The groups A and B were imbalanced for tumor size (*d* = 0.392), T (*d* = 1.128), N (*d* = 1.340), M (*d* = 1.456), mean number of positive lymph nodes (*d* = 0.907), and LNR (*d* = 0.889). Before the balancing, the risk of death was higher in group B than in A (4.3; 2.5 to 7.4). After reweighting, all large differences were eliminated (*d* < 0.200). In high-risk patients, the risk of death was higher in patients who underwent surgery alone than those who received perioperative chemotherapy (HR 0.5; 0.2 to 1.3) without statistical significance (*p* = 0.187). In low-risk patients, the risk of death was similar (HR 1.1; 0.3 to 3.3).

**Conclusion:**

Perioperative chemotherapy could provide some marginal advantages to high-risk patients.

## Introduction

Goblet cell carcinoma (GCC) is a rare appendiceal tumor composed of cells with secretory phenotypes, including goblet cells, endocrine cells, and Paneth cells [1]. The biological behavior ranges from indolent to aggressive, depending on the extent of the adenocarcinoma component [2, 3]. The diagnosis is often causal during emergent appendectomy and routinely requires a second look with right hemicolectomy (RHC) [4]. Indeed, the only curative treatment available for GCCs is radical surgery. However, the overall survival (OS) depends on the pathological stage ranging from 14 to 86% at 5 years [5]. The benefits of adjuvant or neoadjuvant chemotherapy combined with radical surgery remain debatable and hardly demonstrable. Indeed, on the one hand, the rarity of this disease makes it challenging to perform prospective comparative studies. On the other hand, the available retrospective studies reported conflicting and questionable results [5–7]. The main limitation of retrospective studies was the evident imbalance for the AJCC stage and tumor size between GCC patients who received surgery alone and those treated with surgery and perioperative chemotherapy [7]. Moreover, the sample size of the second group is often small, making it difficult to obtain credible information using classical Cox regression multivariate analysis [8, 9]. The present study aims to fill this gap by adjusting all confounding factors with a novel method called the entropy balancing approach [10]. For this purpose, patients with GCCs, extracted from the Surveillance, Epidemiology, and End Results (SEER) study database, were used.

## Materials and methods

The SEER registry was explored using the SEER*stat software (https://seer.cancer.gov) to include all patients diagnosed with resected GCC. Records were selected by histology according to ICD-O-3 diagnosis code 8243/3 (goblet cell carcinoid) from the “Incidence—SEER 18 Regs Research Data + Hurricane Katrina Impacted Louisiana Cases (with additional treatment fields), Nov 2018 Sub (1975–2016 varying)” based on the November 2018 submission database.

The exclusion criteria were (1) procedures different from surgical resection such as local or endoscopic excision; (2) only appendectomy; (3) non-surgical treatments; (4) absence of survival data; and (5) absence of information about chemotherapy. The following data were analyzed: age, gender, tumor size, staging according to the American joint committee on Cancer (AJCC) 7th edition [11], TNM staging, resection of the primary tumor, grading, number of regional nodes examined, and number of positive regional nodes (i.e., confirmed by pathology to contain tumor cells).

The overall survival (OS) was calculated from diagnosis to death. Permission to access the SEER database was granted on 19/03/2020 with authorization number 21495-Nov2018.

The analysis was carried out in three steps. First, the patients were allocated into two groups: patients treated with surgery alone (group A) and those who underwent surgery plus perioperative chemotherapy (group B). The two groups were compared without correcting the selection bias. In the second step, a virtual group A was generated using the entropy balancing approach. Virtual group A assumed group B’s demographic, clinical, and pathological characteristics. In other words, we simulated a well-balanced randomized control trial (RCT) that enrolled the typical patient candidate for perioperative chemotherapy according to the current clinical practice (model 1). In the third step, a “symmetric” procedure was carried out: the group B patients were transformed into a virtual cohort similar to group A. In other words, a well-balanced RCT between surgery alone vs. surgery plus perioperative chemotherapy was simulated. However, in this RCT, we tested the efficacy of chemotherapy in patients who did not undergo it in clinical practice.

### Statistical analysis

Data were reported in percentages or mean and standard deviation (SD). The survival was described using restricted mean survival because this measure permits the description of the survival data even if the median is not reached. Differences between the groups were measured using standardized differences (*d* value). A *d* value ≤ 0.2 indicates a percentage of the non-overlap population ≤ 15% (small difference); a *d* value > 0.2 and ≤ 0.5 (medium difference) means that the percentage of the non-overlapped population was > 15% but ≤ 33%; a *d* value > 0.5 to 0.8 (large difference) indicates a percentage of non-overlap population > 33%. All endpoints were reported for the unmatched and matched populations. A preliminary multivariate Cox regression was made to explore the hypothesis that some factors could influence overall survival. If this hypothesis is validated, a correction of the imbalance between the two groups was performed by the entropy balancing approach. The entropy balancing, described by Hainmueller et al. [10], was applied to eliminate the selection bias due to retrospective design.

In contrast to other preprocessing methods, such as propensity score matching, entropy balancing involves a reweighting scheme that directly incorporates covariate balance into the weight function that is applied to the sample units. In other words, entropy balancing not eliminates the marginal cases, but it reweights the characteristics of patients (covariates) of one group to be similar to the comparative one. This recalibration of the unit weights effectively adjusts for systematic and random inequalities in representation [10]. It should be noted that, in the table, the frequency and percentage of discrete variables did not change after reweighting in virtual groups. However, the weight of each class within the same variable changed. The survival curves were built by performing proportional hazards regression. The statistical analyses were computed using STATA software (StataCorp. 2011. College Station, TX: StataCorp LP). Entropy balance was performed with the “ebalance” module.

## Results

**Fig. 1:**
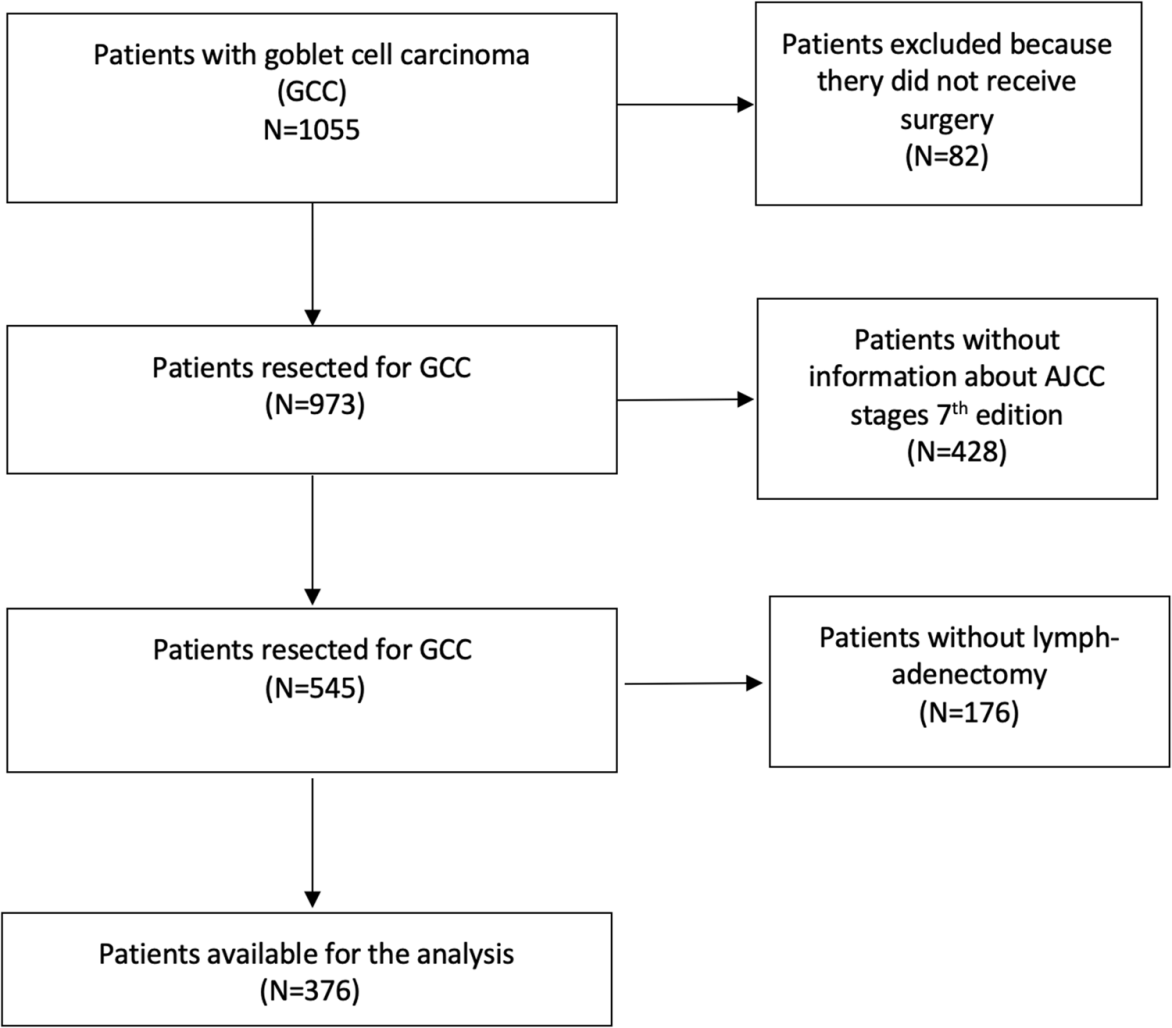
Flow chart of patients’ selection for the analysis

Starting from 1055 records, only 376 patients were included in the final analysis: 309 (82.1%) received surgery alone (group A) and 67 (17.8%) surgery plus perioperative chemotherapy (group B). All patients included were treated after 2011. The selection process of patients is described in Fig. 1.

### Before reweighting

**Fig. 2:**
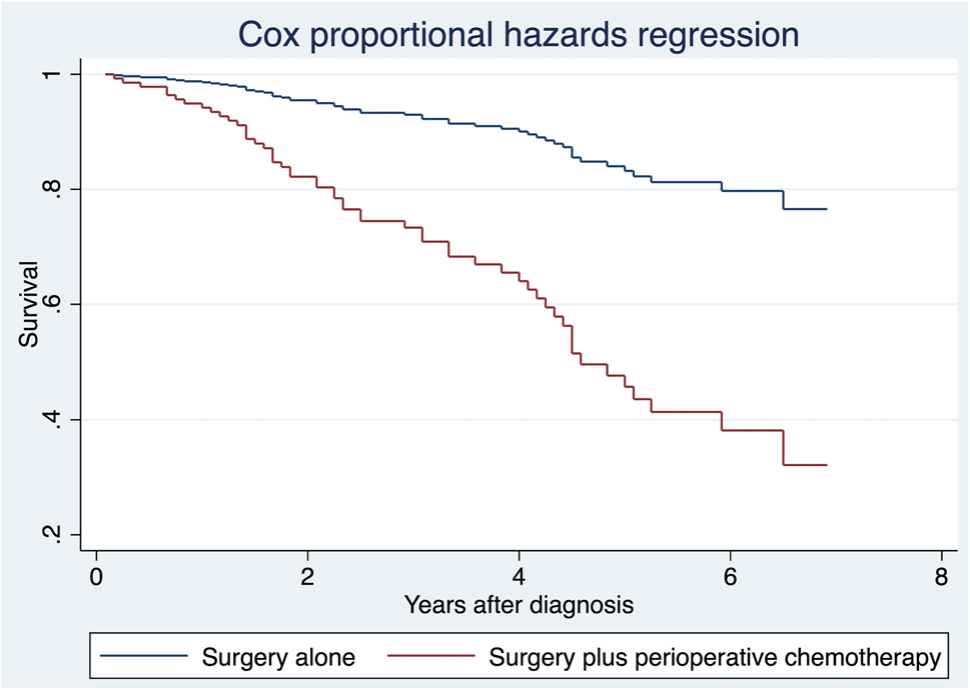
Cox proportional hazards regression before entropy balancing

The baseline characteristics of the two groups are compared and reported in Table 1. Before re-weighting, the patients who received perioperative chemotherapy have larger tumors (*d* = 0.392), higher T status (*d* = 1.128), more frequently nodal (*d* = 1.340), or distant metastases (*d* = 1.456), or poorly differentiated tumors (*d* = 0.687). Moreover, patients in group B have a higher mean of positive lymph nodes (*d* = 0.907) and higher LNR (*d* = 0.889) than group A. The risk of death was higher in patients who received perioperative therapy than in those treated with surgery alone (HR 4.2, 2.5 to 7.4; *p* < 0.001). The multivariate analysis showed that several covariates could influence overall survival (Table 2), justifying the reweighting. Particularly, overall survival was decreased by age (HR 1.1, 0.9 to 1.2; *p* = 0.088), lymph nodal metastases (HR 1.8, 0.9 to 3.4; *p* = 0.062), distant metastases (HR 1.7, 1.2 to 2.4; *p* < 0.001), high LNR (HR 46.1, 6.5 to 326.1; *p* < 0.001) (Fig. 2).

**Table 1:** The baseline characteristics of the two groups

*Parameters*	*Before weighting*			*Model 1*		*Model 2*	
	*Surgery alone* *(N* = *309)*	*Surgery plus adjuvant chemotherapy (N* = *67)*	*|d value|*	*“Virtual”* *Only surgery*	*|d value|*	*“Virtual”* *Surgery plus adjuvant chemotherapy*	*|d value|*
*Gender*					0		0
*Male*	150 (48.5)	31 (46.3)	0.050	150 (48.5)		31 (46.3)	
*Female*	159 (51.5)	36 (53.7)		159 (51.5)		36 (53.7)	
*Age, years*	58.6 ± 12.3	56.6 ± 9.4	0.170	56.6 ± 13.3	0	58.7 ± 8.6	0
*Tumor size*	28.6 ± 20.7	36 ± 17.8	0.392	36.5 ± 19.4	0	28.9 ± 12.6	0.009
*T according to 7th AJCC Ed*					0.003		0.009
*Tis*	3 (1)	0 (0)	1.128	3 (1)		0 (0)	
*T1*	21 (6.8)	1 (1.5)		21 (6.8)		1 (1.5)	
*T2*	29 (9.4)	0 (0)		29 (9.4)		0 (0)	
*T3*	216 (69.9)	28 (41.8)		216 (69.9)		28 (41.8)	
*T4a*	31 (10)	18 (26.9)		31 (10)		18 (26.9)	
*T4b*	9 (2.9)	20 (29.9)		9 (2.9)		20 (29.9)	
*N according to 7th AJCC Ed*					0.092		0.015
*N0*	279 (90.3)	28 (41.8)	1.340	279 (90.3)		28 (41.8)	
*N1*	22 (7.1)	27 (40.3)		22 (7.1)		27 (40.3)	
*N2*	8 (2.6)	12 (17.9)		8 (2.6)		12 (17.9)	
*M according to 7th AJCC Ed*					0		0.078
*M0*	301 (97.4)	38 (56.7)	1.456	301 (97.4)		38 (56.7)	
*M1a*	1 (0.3)	2 (3)		1 (0.3)		2 (3)	
*M2b*	3 (1)	23 (34.3)		3 (1)		23 (34.3)	
*Mnos*	4 (1.3)	4 (6)		4 (1.3)		4 (6)	
*RHC*			0.095		0		0
*Typical*	84 (27.2)	16 (23.9)		84 (27.2)		16 (23.9)	
*Atypical*	225 (72.8)	51 (76.1)		225 (72.8)		51 (76.1)	
*Grading*			0.687		0		0.073
*Unspecified*	172 (55.7)	28 (41.8)		172 (55.7)		28 (41.8)	
*Well-differentiated*	67 (21.7)	7 (10.4)		67 (21.7)		7 (10.4)	
*Moderately differentiated*	54 (17.5)	8 (11.9)		54 (17.5)		8 (11.9)	
*Poorly differentiated*	16 (5.2)	24 (35.8)		16 (5.2)		24 (35.8)	
*Mean harvested lymph nodes*	16.5 ± 10	17.8 ± 8.6	0.110	17.8 ± 7.9	0	17.8 ± 8.6	0.006
*Mean positive lymph nodes*	0.3 ± 1.7	2.4 ± 4.2	0.907	0.3 ± 0.1	0	2.4 ± 4.2	0
*LNR*	0.02 ± 0.11	0.16 ± 0.19	0.889	0.16 ± 0.18	0	0.16 ± 0.19	0.006

Legend: A d -value $\leq$ 0.2 indicates a percentage of the non-overlap population $\leq$ 15% (small difference); a d -value >0.2 and $\leq$ 0.5 (medium difference) means that the percentage of the non-overlapped population was > 15% but $\leq$ 33%; a *d *value >0.5 to 0.8 (large difference) indicates a percentage of non-overlap population > 33%; *AJCC, *American Joint Cancer Committee*; Ed,* Edition*; LNR,* lymph nodes ratio

**Table 2:** Multivariate models

*Parameters*	*Multivariate model*	
	*OR (95 CI)*	*p value*
*Gender*		
*Male*	Referent	
*Female*	1.1 (0.6 to 1.9)	0.934
*Age, years (for each year)*	1.1 (0.9 to 1.1)	0.088
*Tumor size (for each mm)*	0.9 (0.9 to 1.1)	0.801
*T according to 7th AJCC Ed (increasing T stage)*	1.3 (0.9 to 1.9)	0.238
*N according to 7th AJCC Ed*		
*No*	Referent	0.062
*Yes*	1.8 (0.9 to 3.4)	
*M according to 7th AJCC Ed*		
*No*	Referent	0.001
*Yes*	1.7 (1.2 to 2.4)	
*RHC*		
*Typical*	Referent	0.934
*Atypical*	0.6 (0.3 to 1.2)	
*Grading (for each grade)*	1.1 (0.8 to 1.4)	0.141
*Harvested lymph nodes*	0.9 (0.9 to 1.1)	0.581
*Positive lymph nodes*	0.9 (0.8 to 1.1)	0.941
*LNR*	46.1 (6.5 to 326.1)	< 0.001
*Perioperative chemotheraphy*	1.1 (0.5 to 2.2)	0.803

*OR*, odds ratio; *CI*, confidence interval; *AJCC,* American Joint Cancer Committee; *Ed*, edition; *LNR,* lymph node ratio

### After reweighting

**Fig. 3:**
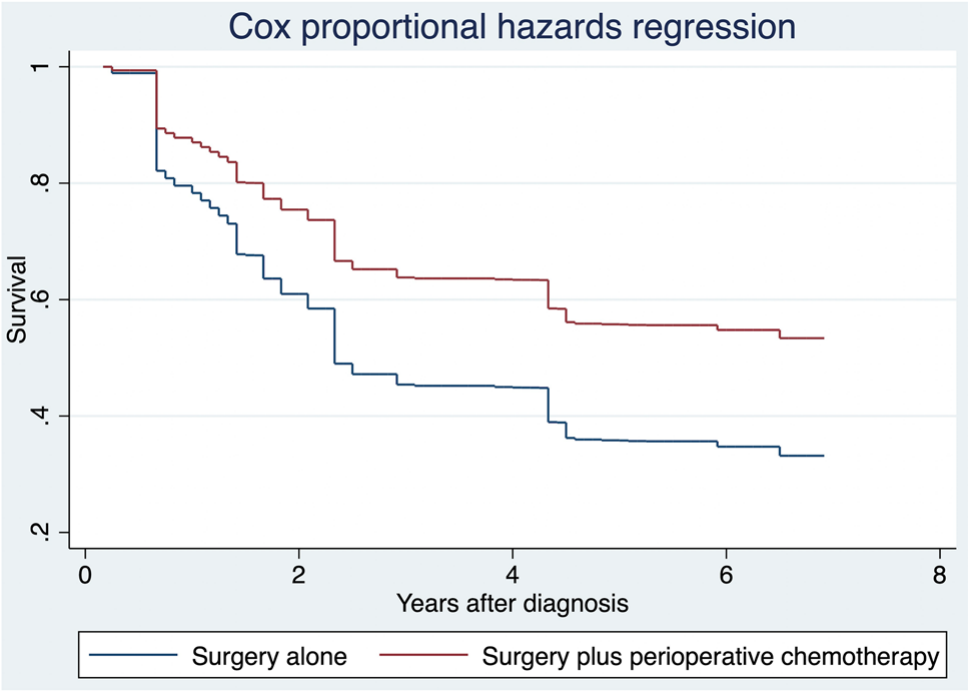
Cox proportional hazards regression after entropy balancing. Legend: The “Surgery alone” group is reweighted to be similar “Surgery plus perioperative chemotherapy” group

**Fig. 4:**
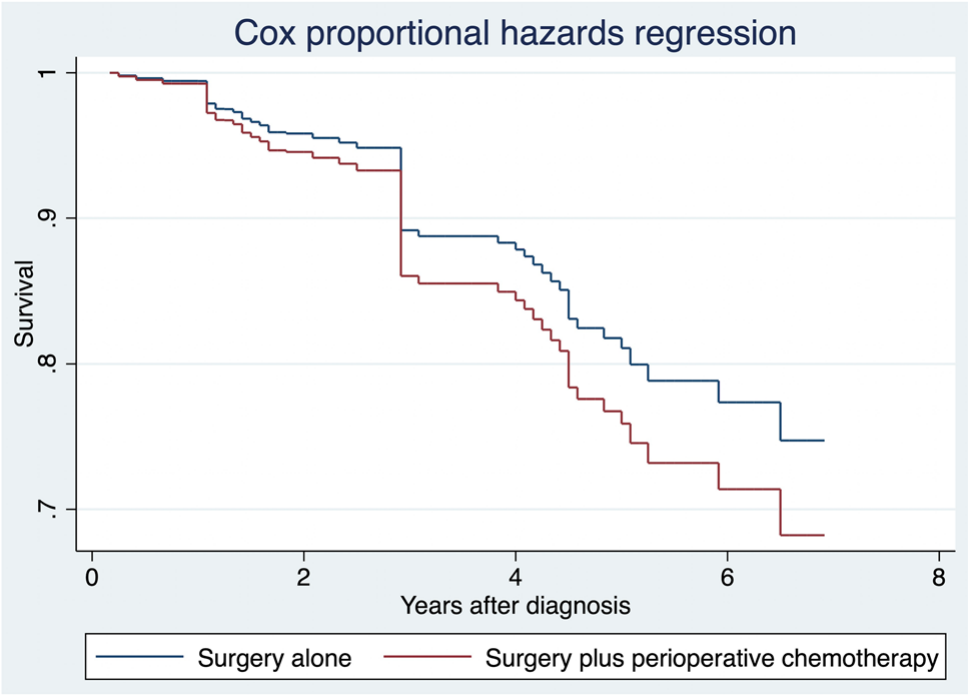
Cox proportional hazards regression after entropy balancing. Legend: The “Surgery plus perioperative chemotherapy” group is reweighted to be similar “Surgery alone”   group

In model 1, all differences between the two groups (B and virtual A) were small (*d* values < 0.200). The risk of death was higher in patients who received surgery alone than in those treated with surgery plus perioperative chemotherapy (HR 0.5, 0.2 to 1.2), even if the results were not statistically significant (*p* = 0.235), as reported in Fig. 3. In model 2, all differences between the two groups (A and virtual B) were small (*d* values < 0.200), as reported in Fig. 4. The risk of death (Table 2) was similar among the two groups (HR 1.3, 0.4 to 5.0; *p* = 0.682).

## Discussion

The present study clarifies the role of perioperative therapy after radical resection of appendiceal GCC. Considering the rarity of GCC, the SEER database was used. The selection process of patients was meticulous in including only patients with adequate information. Indeed, all cases included in the final analysis were diagnosed after 2011. The preliminary multivariate Cox regression demonstrated that selection bias could influence the overall survival and should be corrected.

Moreover multivariate model underlined that perioperative chemotherapy could be unnecessary in some patients having GCC. The methodology used to correct the selection bias is original and permits avoiding the imbalance problem due to allocation treatment without randomization. The entropy balancing is different from propensity score matching (PSM). Indeed, PSM involves simply discarding units to improve the covariate balance between the treatment and control group with a loss of sample size and information. Entropy balancing permits maintaining the marginal cases, reweighting only the covariates of one group to be equal to the comparative one. This recalibration of the unit weights effectively adjusts, in an entropic way, for systematic and random inequalities in representation. De facto, entropy balancing allows the simulation of two well-balanced randomized trials. The first model answered a simple question: “what would have happened if we had not exposed our high-risk patients to perioperative chemotherapy?”. In other words, we simulated an RCT, never performed and challenging to design, in which the population target was the same that, in current clinical practice, received chemotherapy: large tumors or high T, grade, rate of lymph node-positive, LNR, and N or M status positive. In this model, chemotherapy combined with radical surgery could provide a marginal survival advantage.

Indeed, the difference was not statistically significant even if the restricted mean survival was superior to the patients who received only surgical treatment. These results are not surprising because all monocentric series demonstrated no advantages in overall survival when adjuvant 5-fluorouracil-based chemotherapy was used in stages III and IV [12, 13]. Moreover, the small series [14] about preoperative or intraoperative chemotherapy and limited to stage IV confirmed the short life expectancy in this setting. By the way, larges studies based on National Cancer Database (NCDB) have conflicting results. Zakka et al. [8], analyzing 619 patients obtained, reported the lacking of advantages for adjuvant chemotherapy when all stages were included.

However, when the subgroup of stage III (*n* = 107) was considered, some survival benefits emerged for those who received chemotherapy. AlMasri et al. [15], using the same NCBD dataset, reached a different conclusion demonstrating that chemotherapy could positively influence OS in all patients independently from the stage. Nonetheless, the main limitation of both analyses [8, 15] was that the judgment was based on underpowered multivariate analysis. Indeed in both series, the low rate of events per participant and the high number of covariates make “at-risk” the results obtained with multivariate models [13]. On the contrary, in our model, a “head to head” comparison was made in well-balanced groups between the two strategies, reducing the risk of overfitting [16]. These results did not surprising. Rossi et al. [2] underlined that the prognosis of advanced appendiceal GCC is more similar to colonic cancer than to low-grade neoplasms such as midgut neuroendocrine tumors.

The second model of the present analysis answered a second question: “what would have happened if we had exposed our low-risk patients to perioperative chemotherapy?”. In other words, we simulated an RCT, which is challenging to design for ethical problems, in which the population target was the same that, in current clinical practice, not received chemotherapy: small tumors or low T, grade, rate of lymph node-positive, LNR, and N or M status negative. The present study confirms that, in this setting, the perioperative chemotherapy was useless, and it did not prolong survival. The futility of adjuvant therapy in stages I–II seems credible because the OS survival rate was very high also without chemotherapy (86% at 5 years) [15]. Moreover, both NANETS [17] and ENETS [18] guidelines recommended only right hemicolectomy in patients with low grades and the absence of nodal and distant metastases.

The present study has some limitations. First, the design is retrospective and based on an institutional database. Thus, the risk of bias due to incorrect registration or classification is present. Moreover, the AJCC stage is changed during the observation time. Nonetheless, the patients were meticulously selected to avoid bias or misinterpretation using stringent criteria. Remarkably, all patients without the 7th edition of the AJCC stage were excluded, and in the final analysis, only patients recently treated were included. Second, the estimates are based on the methodological approach of entropy balancing. This statistical instrument reduces but does not entirely solve the problem of causal inference due to the lacking of random allocation.

Nonetheless, it could provide helpful information in a setting where an RCT could be difficult to design or not ethical. Indeed, the overall incidence of GCCs is very low (0.01–0.05 per 100,000 persons annually), and the quote of patients (stages III–IV) who could have some benefits from the chemotherapy represents 10–15%. On the other hand, performing an RCT with patients in stages I–II could be unethical, considering the increased risk of adverse events without any hypothesis of survival advantages. Third, it is impossible to distinguish between pre-, intra-, or post-operative chemotherapy within SEER data. However, considering the current clinical practice, the patients who underwent preoperative or intraoperative chemotherapy were limited, and all were in stage IV. Finally, the SEER database did not provide information about chemotherapy regimens and schedules. Moreover, it is impossible to extract the adverse effects rates and severity due to the chemotherapy and long-term quality of life of patients. These factors could be crucial in evaluating perioperative therapy's risk/benefit ratio.

In conclusion, perioperative therapy seems to have marginal benefits in GCCs resected in stages III and IV. Chemotherapy should be performed only in selected cases. In patients with GCCs in stages I–II, the RHC should be considered curative, and no adjuvant chemotherapy should be planned. Further and extensive studies should be conducted to confirm our results.
